# Tissue Expression Analysis, Cloning, and Characterization of the 5′-Regulatory Region of the Bovine *LATS*1 Gene

**DOI:** 10.3389/fvets.2022.853819

**Published:** 2022-05-26

**Authors:** Dawei Wei, Sayed Haidar Abbas Raza, Xingping Wang, Rajwali Khan, Zhaoxiong Lei, Guijie Zhang, Jiupan Zhang, Zhuoma Luoreng, Yun Ma, Muna O. Alamoudi, Bandar Hamad Aloufi, Ahmed Mohajja Alshammari, Ayman Hassan Abd El-Aziz, Majid Alhomrani, Abdulhakeem S. Alamri

**Affiliations:** ^1^School of Agriculture, Ningxia University, Yinchuan, China; ^2^Key Laboratory of Ruminant Molecular Cell Breeding, Ningxia Hui Autonomous Region, Yinchuan, China; ^3^College of Animal Science and Technology, Northwest A&F University, Yangling, China; ^4^Department of Livestock Management, Breeding and Genetics, The University of Agriculture, Peshawar, Pakistan; ^5^Institute of Animal Science, Ningxia Academy of Agriculture and Forestry Sciences, Yinchuan, China; ^6^Department of Biology, College of Science, University of Hail, Hail, Saudi Arabia; ^7^Animal Husbandry and Animal Wealth Development Department, Faculty of Veterinary Medicine, Damanhour University, Damanhour, Egypt; ^8^Department of Clinical Laboratories Sciences, The Faculty of Applied Medical Sciences, Taif University, Taif, Saudi Arabia; ^9^Centre of Biomedical Sciences Research (CBSR), Deanship of Scientific Research, Taif University, Taif, Saudi Arabia

**Keywords:** *LATS*1 gene, expression, core promoter, transcription, factor

## Abstract

As a member of the large tumor suppressor (*LATS*) gene family, *LATS*1 plays an important role in regulating muscle growth and development. In this study, we determined the distinct exhibit patterns of tissue expression of bovine *LATS*1. Further, we determined the functional proximal minimal promoter of bovine *LATS*1 and identified the key transcription factors in the core promoter region to elucidate its molecular regulation mechanism. The results showed that bovine *LATS*1 was highly expressed in the *longissimus thoracis* and upregulation in infancy muscle. An electrophoretic mobility shift assay (EMSA) and chromatin immunoprecipitation (ChIP) assay in combination with site-directed mutation and small interfering RNA (siRNA) interference demonstrated that myogenic differentiation 1 (Myod1) and myocyte enhancer factor 2A (MEF2A) binding in the core promoter region (−298/−123 bp) play important roles in the transcriptional regulation of the bovine *LATS*1 promoter. Taken together, these interactions provide insight into the regulatory mechanisms of *LATS*1 transcription in mediating skeletal muscle growth in cattle.

## Introduction

The beef cattle industry in China has surely got a rapid development momentum, although it started late and has been at a low level in the past decade. At present, beef consumption in China is in short supply and the price continues to run at a high level. Therefore, China has to import a large amount of beef from abroad. In 2020, the beef import volume reached 2.12 million tons, ranking first in the world. It is predicted that in the next 5 years, the annual consumption of beef in China will exceed 10 million tons. According to the current production level, there is a gap of at least 3 million tons. Therefore, there is still much need for the development of the beef cattle industry in China (1).

Studies have shown that the formation of skeletal muscle tissue in animals is not only closely related to genetic, environmental, and nutritional levels, but also more dependent on gene control (2, 3). Therefore, it is of great importance to improve the growth and development of skeletal muscle and meat quality by gene screening and functional identification (4). As one of the main members of Hippo pathway, the large tumor suppressor 1 (*LATS*1) gene plays an important role in the formation of tissues and organs and the proliferation and differentiation of embryonic stem cells (5, 6). In addition, the Hippo-yes-associated protein (YAP)-*LATS*1 signaling pathway has emerged as one of the critical conserved pathways that regulate cell proliferation and apoptosis in response to environmental and developmental signals (6–8). Phosphorylation of *LATS*1/2 is required for activation of YAP that performs roles in the regulation of maintenance of stem cell pluripotency, tumorigenesis, and organ size (9, 10). It has been discovered that in many molecules, *LATS*1 is involved in the Hippo pathway, but mechanisms governing activation of these proteins and activation of the pathway, in general, are currently not clear. At present, the study on *LATS*1 is mainly on the mechanism of tumor cell proliferation (11–13). At present, there is no report on the *LATS*1 gene regulating muscle growth and development of cattle.

In our previous study, we identified the *LATS*1 gene related to growth traits in Qinchuan cattle. We speculate that *LATS*1 plays an important regulatory role in the process of muscle cell differentiation, whereas the regulation mechanism of *LATS*1 promoter activity during differentiation, especially for transcriptional, is still unclear. Herein, we analyzed the molecular mechanisms of *LATS*1 gene regulation *via* the promoter region. In addition, the relative messenger RNA (mRNA) expression pattern of bovine *LATS*1 in tissue was determined. Our results provide a solid basis for further insight into the transcriptional regulatory mechanisms for *LATS*1 in mediating skeletal muscle growth in cattle.

## Materials and Methods

### Ethics Statement

All the animal operating procedures are in line with the China Council on Animal Care, as well as the standards set by the Ningxia University of experimental animal management practices.

### Quantitative PCR Analysis of Gene Expression Patterns

To compare gene expression level between different tissues and temporal expression of bovine *LATS*1 gene, twelve tissues (heart, liver, spleen, lung, kidney, small intestine, large intestine, abdominal fat, *longissimus thoracis*, rumen, testis, and brain tissue) were obtained from three adult Qinchuan cattle. Further, we collected *longissimus thoracis* samples during six developmental stages of male Qinchuan cattle, namely, 0, 6, 12, 24, and 36 months after birth. We assayed gene expression in triplicate for each sample and normalized the data using the housekeeping gene glyceraldehyde-3-phosphate dehydrogenase (GAPDH). The quantitative primers of *LATS*1 and GAPDH were designed and given in Table 1. Briefly, the total RNA in different tissues was extracted using the MiniBEST Universal RNA Extraction Kit (TaKaRa) and complementary DNA (cDNA) was synthesized from 1 μl of the extracted RNA using the PrimeScript™ RT Reagent Kit (TaKaRa) according to the manufacturer's protocols. 2 μl of the reverse transcription reaction of different tissues [equivalent to 20 ng total RNA per 20 μl quantitative PCR (qPCR) reaction] was used to determine relative expression of *LATS*1 in 2-step qPCR by using the Real-Time PCR Detection System (Bio-Rad, USA). The relative expression levels of the target mRNAs were calculated using the 2^−Δ*ΔCt*^ method (14).

**Table 1:** Primers utilized in this study.

**Item**	**Primer name**	**Primer sequence(5'to3')**	**Tm (**°**C)**	**Product length (bp)**	**Amplified region**	**Accession numbers**
RT-PCR	GAPDH	F:CCAACGTGTCTGTTGTGGAT	60.0	80	320–521	NM_001034034.2
		R:CTGCTTCACCACCTTCTTGA				
	*LATS*1	F:GTTGGTAGGACAGCCGCCTTTC R:GCTTGCGGTGGGATGTGAAGAG	60.0	96	3,310–3,406	NM_001192866.1
Promotercloning	*LATS*1-PF/PR	F:CGACTGAGCGACTGAACTGAAG R:GCCGCCATTTTGCCTTTCACTCG	65.5	1,950	−1,783/+167	NC_037336.1
	*LATS*1-P1	F:*CGGGGTACC*CGACTGAGCGACTGAACTGAAG	64.0	1,950	−1,783/+167	
	*LATS*1-P2	F:*CGGGGTACC*TACACCCGAAACTAGCACAA	61.5	1,616	−1,449/+167	
	*LATS*1-P3	F:*CGGGGTACCC*TTCACAGGAATAAACCTCTT	63.5	1,316	−1,149/+167	
	*LATS*1-P4	F:*CGGGGTACC*GGGTCATATCTTCTACAGACT	65.0	1,004	−837/+167	
	*LATS*1-P5	F:*CGGGGTACC*CAAAGGAGCTCTGTACAATG	61.5	722	−555/+167	
	*LATS*1-P6	F:*CGGGGTACC*AGTGCTAAGCAAAATTCTAG	62.0	465	−298/+167	
	*LATS*1-P7	F:*CGGGGTACC*AGACACTGAAATACTTGAGT	59.5	290	−123/+167	
	*LATS*1-R	R:*CCCAAGCTT*GCCGCCATTTTGCCTTTCACTCG			+167	
Site-mutand EMSA	Myod1 forward	AGAGGGCAACTCA*CTGC*TGTTTCCTTCTGAGAAA		
	Myod1 reverse	TTTCTCAGAAGGAAACA*GCAG*TGAGTTGCCCTCT		
	mMyod1 forward	AGAGGGCAACTCA*AAAA*TGTTTCCTTCTGAGAAA		
	mMyod1 reverse	TTTCTCAGAAGGAAACA*TTTT*TGAGTTGCCCTCT		
	MEF2A forward	GCATCTTTTATTCTA*TATT*CGGCCACAGAAAGAG		
	MEF2A reverse	CTCTTTCTGTGGCCG*AATA*TAGAATAAAAGATGC		
	mMEF2A forward	GCATCTTTTATTCTA*AAAA*CGGCCACAGAAAGAG		
	mMEF2A reverse	CTCTTTCTGTGGCCG*TTTT*TAGAATAAAAGATGC		
ChIP	ChIP-Myod1	F: GCAAAATTCTAGTTATTTCAAAGTC R: TAAAAGATGCGTATCCGAAGG	126	−290/−165	
	ChIP-MEF2A	F: ACTCCTTCGGATACGCATCTT R: CCTCCTCCCAAGCCCACTCAA	100	−188/−89	
	ChIP-control	F: TGGTTATACAAGCTCTTCAGA R: CAAGTTGGCATTAATAGGTCT	137	18,978/19114	3	

*The forward (F) and reverse (R) primers for promoter cloning and the italicized letters indicate the enzyme cutting sites (KpnI and BglII). The underlined bases are core putative transcription factor binding sites*.

### Cloning and Sequence Analysis

We obtained 5′-regulatory region sequence −1,783/+167 of bovine *LATS*1 according to the sequence information in GenBank (NC_037345, from 22618410 to 22620410). PCR amplifications were performed with genomic DNA using Qinchuan cattle blood as a template with the corresponding primers PF and PR (Table 1). Nonetheless, the PCR product yielded 5'-regulatory region sequences as verified by analysis. The structure and the constitution of amino acid of bovine *LATS*1 gene were analyzed by a series of bioinformatics software. Further, the potential transcription factor (TF) binding sites and Cytosine Guanine (linear dinucleotide) (CpG) islands were analyzed using Genomatix (http://www.genomatix.de/) (15) and MethPrimer (http://www.urogene.org/methprimer/) (16), respectively. The threshold for screening potential transcription factor-binding sites was >0.9.

### Luciferase Reporter Constructs

Seven specific primers that included the restriction sites of the KpnI and BglII were designed to amplify the unidirectional deletions of the bovine *LATS*1 promoter. The PCR products were processed by gel extraction, purification, and were digested with the restriction enzyme KpnI and BglII (New England BioLabs Incorporation, USA) following the manufacturer's protocol. Then, the fragments were ligated into the luciferase reporter construct pGL3-basic vector digested with the same restriction enzymes (KpnI and BglII), respectively. These plasmids were named pGL3-*LATS*1-P1, pGL3-*LATS*1-P2, pGL3-*LATS*1-P3, pGL3-*LATS*1-P4, pGL3-*LATS*1-P5, pGL3-*LATS*1-P6, and pGL3-*LATS*1-P7.

### Cells Culture and Transfection

Qinchuan cattle myoblast cells (QCMCs) were isolated under a dissection microscope from the Qinchuan fetal bovine muscle samples, as described previously (17). QCMCs were cultured and grown in triplicate 24-well plates until reaching a density of 70% at 37°C and 5% CO_2_. Briefly, 800 ng of the seven pGL3-*LATS*1-P1 to pGL3-*LATS*1-P7 luciferase construct were co-transfected with 10 ng of pRL-TK normalizing vector by using Lip3000 (Invitrogen) according to the manufacturer's instructions. After 6 h post-transfection, the QCMCs culture medium [10% fetal bovine serum (FBS) + 90% Dulbecco's Modified Eagle Medium (DMEM)] was replaced with differentiation medium (2% horse serum + 98% DMEM) to induce differentiation of the myoblasts into my tubes as parallel controlled. The pGL3-basic vector was used as a negative control. Forty-eight hours after transfection, cells lysates were collected and followed by quantification of firefly and *Renville* luciferase expression by performing dual-luciferase activity assays in the NanoQuant Plate™ (Tecan, Infinite M200PRO).

### Site-Directed Mutagenesis

The potential TF-binding sites were analyzed using the Genomatix software and mutated with the corresponding primers (Table 1) using the Fast-Directed Mutagenesis Kit (TaKaRa) according to the manufacturer's protocol. Briefly, 800 ng of the mutation vectors were identified and co-transfected into QCMCs with 10 ng of pRL-TK normalizing vector. The wild-type plasmid served as a negative control. The specific operation method was the same as above.

### Myogenic Differentiation 1 and Myocyte Enhancer Factor 2A Knockdown

Specific small interfering RNAs (siRNAs) against target TFs myogenic differentiation 1 (Myod1) or myocyte enhancer factor 2A (MEF2A) were synthesized and co-transfected with pGL3-*LATS*1 core promoter plasmid vector, respectively. The siRNA (UUCCGAACGUGACGUTT) served as a negative control. Briefly, 800 ng of pGL3-*LATS*1 core promoter plasmid and 50 nM (final concentration) of each siRNA duplex were co-transfected into QCMCs using Lip3000 according to the manufacturer's instructions. Twenty-four hours later, luciferase activity was measured using the dual-luciferase reporter assay (E1910, Promega).

### Electrophoretic Mobility Shift Assays

Protein–DNA interactions were evaluated by electrophoretic mobility shift assays (EMSAs) (Thermo Fisher Scientific), as previously described (18). Briefly, 200 fmol of 5'-biotin-labeled Myod1 probe (Table 1) was incubated with 10 μg QCMCs nuclear extracts, 2 μl of 10X binding buffer, and 1 μl of poly (dI-dC) in a volume of 20 μl incubated for 10 min at 37°C. For supershift and competition assays, 2 μg of the anti-Myod1 (sc-31940, Santa Cruz Biotechnology) or anti-MEF2A (EP1706Y, Abcam) antibody or an excess of the respective unlabeled double-stranded oligonucleotides was pre-incubated with the indicated nuclear extract proteins for 10 min. Afterward, samples were resolved on 5% native 0.5X TRIS-boric acid running buffer-polyacrylamide gel electrophoresis and analyzed by the molecular imager ChemiDoc™ XRS+ System (Bio-Rad).

### Chromatin Immunoprecipitation Assay

Chromatin immunoprecipitation (ChIP) assays were used to analyze Myod1 and MEF2A binding to the *LATS*1 gene promoter *in vivo* using the SimpleChIP^®^ Enzymatic Chromatin IP Kit (CST, Massachusetts, USA). For immunoprecipitation, protein–DNA complexes from QCMCs were digested with micrococcal nuclease into fragments of ~150–900 bp in length. The suspension was split into two parts: one of them was clarified and collected with ChIP dilution buffer as an input. Further, another was immunoprecipitated using 4 μl of anti-Myod1 (sc-31940, Santa Cruz Biotechnology) or anti-MEF2A (EP1706Y, Abcam) antibody with gentle mixing overnight at 4°C. The immunoprecipitated products were isolated with protein G agarose beads for ChIP-PCR analysis. The reaction was performed on a number of identified gene targets to confirm specific DNA enrichment vs. ChIP negative control sample and was processed as previously described (19, 20). The percent input was calculated as follows: % Input =2^[−Δ*Ct*(*Ct*[*ChIP*]−(*Ct*[*Input*]−*Log*2(*InputDilutionFactor*)))]^ (21). Normal rabbit immunoglobulin G (IgG) and the intragenic DNA fragment of *LATS*1 exon 3 were used as negative controls.

### Statistical Analysis

Data are expressed as mean ± SD from three independent experiments. The levels of gene expression based on three same feeding conditions by individuals and one-way ANOVA followed by post-ANOVA were used to detect significant differences. Different uppercases and lowercases represent *p* < 0.01 and *p* < 0.05, respectively. The luciferase assays and ChIP based on three independent experiments and the two-tailed Student's *t*-test were used to detect significant differences. “^*^” *p* < 0.05 and “^**^” *p* < 0.01.

## Results

### Detection of Large Tumor Suppressor 1 Expression Pattern

**Figure 1:**
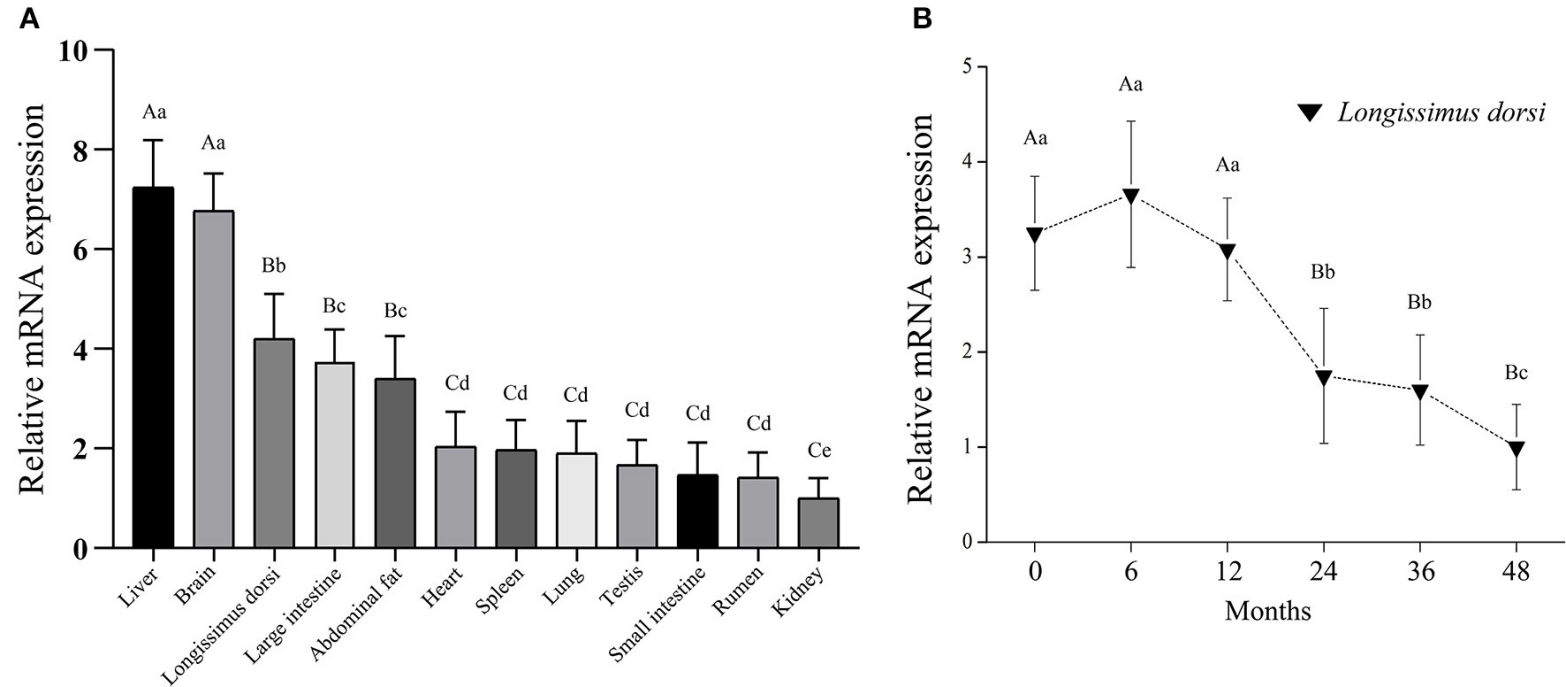
Expression pattern analysis of the bovine large tumor suppressor 1 (*LATS*1). **(A)** Analysis of the bovine *LATS*1 expression pattern in tissues and organs. **(B)** Expression pattern of the bovine *LATS*1 messenger RNA (mRNA) in *longissimus thoracis* at different developmental stages. The value of each column represents the mean ± SD of three independent experiments. Different uppercases and lowercases represent *p* < 0.01 and *p* < 0.05, respectively.

The distribution of bovine *LATS*1 mRNA was determined through qPCR using cDNA from 12 different tissues (Figure 1A). The expression of *LATS*1 gene was significantly higher (*p* < 0.01) in the liver and brain than that in other tissues and it was highly (*p* < 0.05) expressed in *longissimus dorsi*, large intestine, and abdominal fat. However, it was lower in heart, spleen, lung, testis, small intestine, rumen, and kidney. To further determine the temporal expression of bovine *LATS*1 gene in six stages of *longissimus thoracis* at 0, 6, 12, 24, 36, and 48 months. The qPCR was processed and the results showed that the relative expression of bovine *LATS*1 gene in *longissimus thoracis* at 0, 6, and 12 months of age was significantly higher (*p* < 0.01) than that during any other stages (Figure 1B). However, the expression level was reduced dramatically after 24 months (Figure 1B). These results suggest that the *LATS*1 gene is closely related to the development of tissues and organs in Qinchuan cattle, especially in the development of brain and muscle tissue.

### Construction of Bovine Large Tumor Suppressor 1 Gene

**Figure 2:**
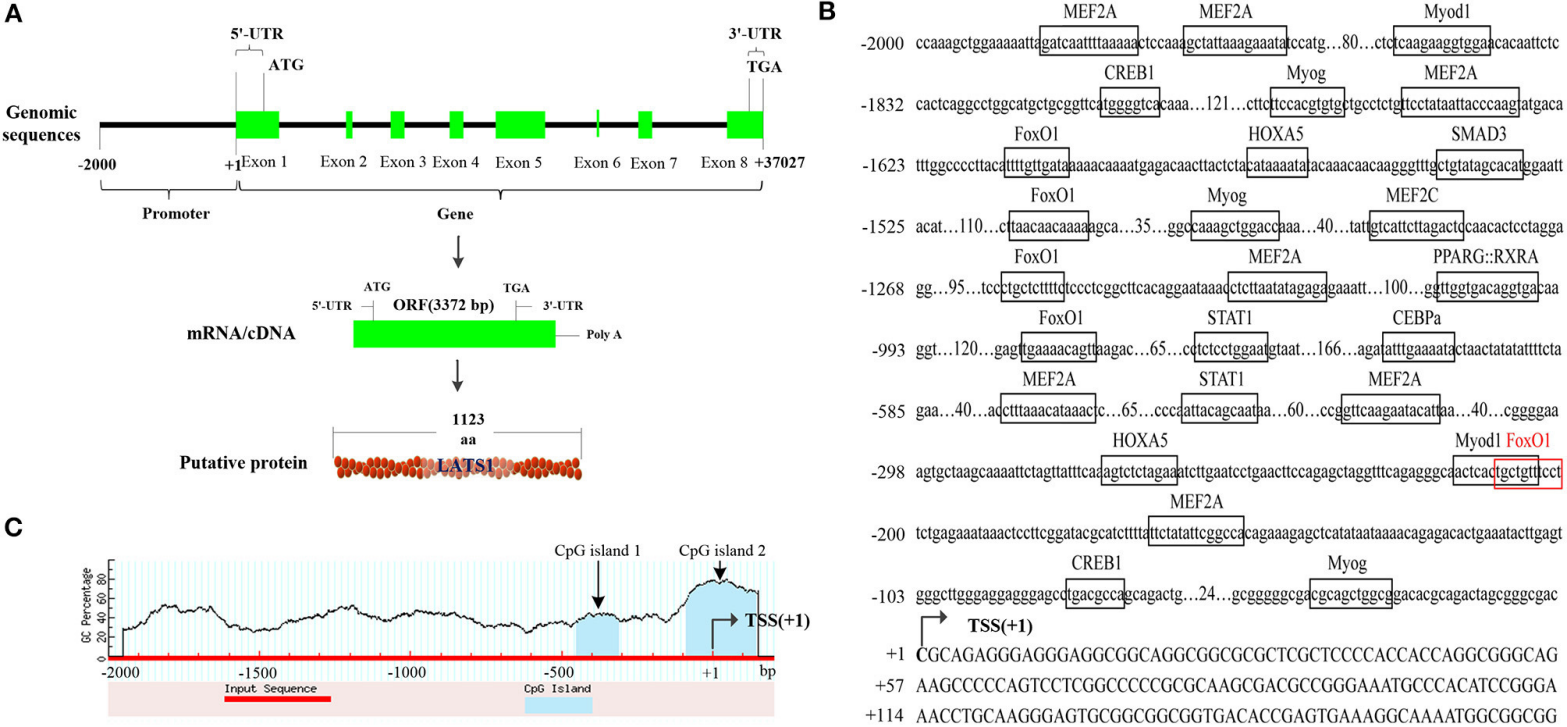
Structural characteristics of the bovine *LATS*1 gene. **(A)** The detailed genomic, mRNA, and protein components are containing the 5′/3′-untranslated region (5′/3′-UTR) and the open reading frame (ORF). **(B)** 5′-regulatory region sequence of the bovine *LATS*1 gene. Arrows mark the transcription initiation sites. The transcription factor binding sites are boxed. **(C)** Dashed lines indicate the GC percentage as represented on the y-axis and the x-axis denotes the bp position in the 5'-untranslated region. Coordinates are given relative to the translational start site (shown as + 1).

Based on the sequence information, bovine *LATS*1 gene was located on chromosome 9 (ENSBTAG00000015076, 86860562-86897588), with a total length of 37,027 bp and including eight exons and seven introns. According to the bovine *LATS*1 DNA sequence, an open reading frame (ORF) of 3,372 bp was verified, which had 1,123 amino acids (aa) (Figures 2A,B) with a chemical formula of C_5, 542_H_8, 646_N_1, 602_O_1, 694_S_43_ and molecular weight of 126.19 KDa. In addition, the promoter region of bovine *LATS*1 gene was cloned from the genome DNA and analyzed the sequence and structural characteristics. The results showed that the bovine *LATS*1 gene promoter region was composed of 647 in A, 544 in T, 368 in G, and 441 in C, resulting in a high GC content of 40.45% and two CpG islands (Figure 2C).

**Figure 3:**
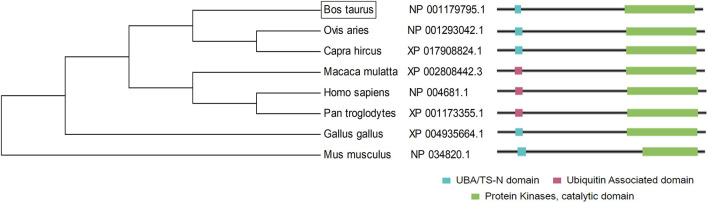
The construction of *LATS*1 protein phylogenetic tree. On the left side, there is the clustering of *LATS*1 protein in different species and on the right side, there is the architecture and characteristics of *LATS*1 protein amino acid length in corresponding species.

Furthermore, the *LATS*1 protein sequences of different species were compared by MEGA software (Figure 3). The results showed that *LATS*1 had high similarity in different species, especially among compound stomach animals such as cattle, sheep, and goat, but also in monogastric animals. This result shows that the function of *LATS*1 gene is very important in different species and is extremely conservative in the process of evolution.

### Determination of Bovine Large Tumor Suppressor 1 Gene Core Promoter

**Figure 4:**
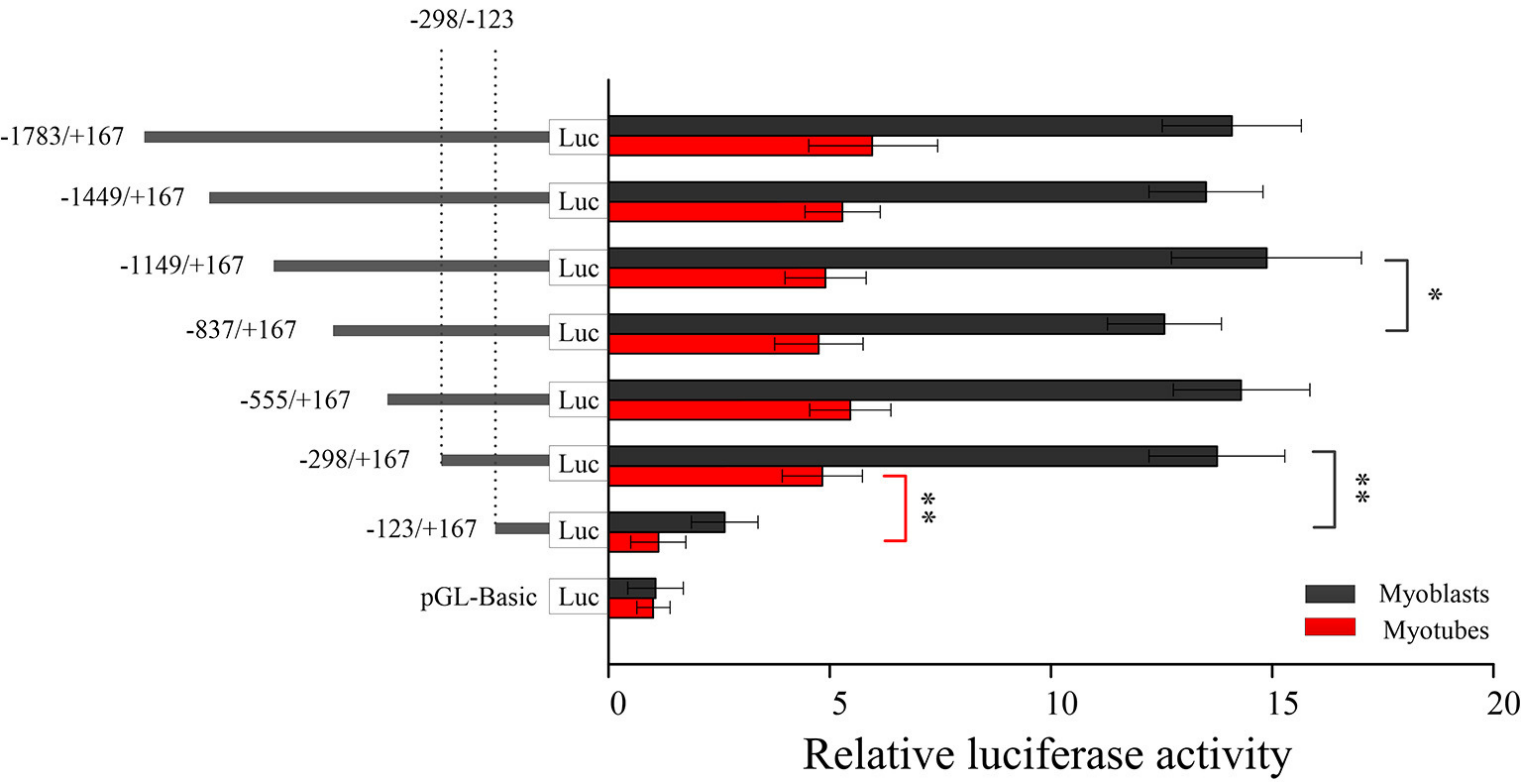
Isolation and analysis of the functional proximal minimal promoter of *LATS*1. A series of plasmids containing 5' unidirectional deletions of the promoter region of the *LATS*1 gene fused in-frame to the luciferase gene were transfected into myoblasts and myotube cells. The results are expressed as the means ± SD in arbitrary units based on the firefly/*Renilla* luciferase activity in triplicate transfections. “*” *p* < 0.05 and “**” *p* < 0.01.

Seven fragments of bovine *LATS*1 gene promoter were obtained using unidirectional deletions PCR and ligated with the pGL3-basic vector to construct the recombinant plasmid. Further, the corresponding plasmid was identified and transfected into myoblast and myotube cell lines by Lip3000 to determine the relative activity of luciferase. The results showed that the p*LATS*1–1,783/+167 activity was significantly higher than that in the pGL3-basic negative control in the two cells (11.8 and 5.3 times higher than that in the negative control group, respectively, Figure 4). It was found that the relative activity of luciferase in p*LATS*1–123/+167 was significantly lower (*p* < 0.01) than that of p*LATS*1–298/+167 both in the myoblast and myotube cell lines when the promoter region −298/−123 bp sequence of *LATS*1 gene was deleted. These results suggest that the 5' upstream 1.7 kb sequence of *LATS*1 gene is a promoter region and has the function of regulating gene transcriptional activity. The promoter region of −298/−123 bp is the core transcriptional region of the *LATS*1 gene. The transcriptional activity of *LATS*1 gene in myoblast was higher than that in the myotube, which indicated that myoblast was more suitable for the determination of *LATS*1 gene transcriptional activity.

### Identifying Key Transcription Factors

**Figure 5:**
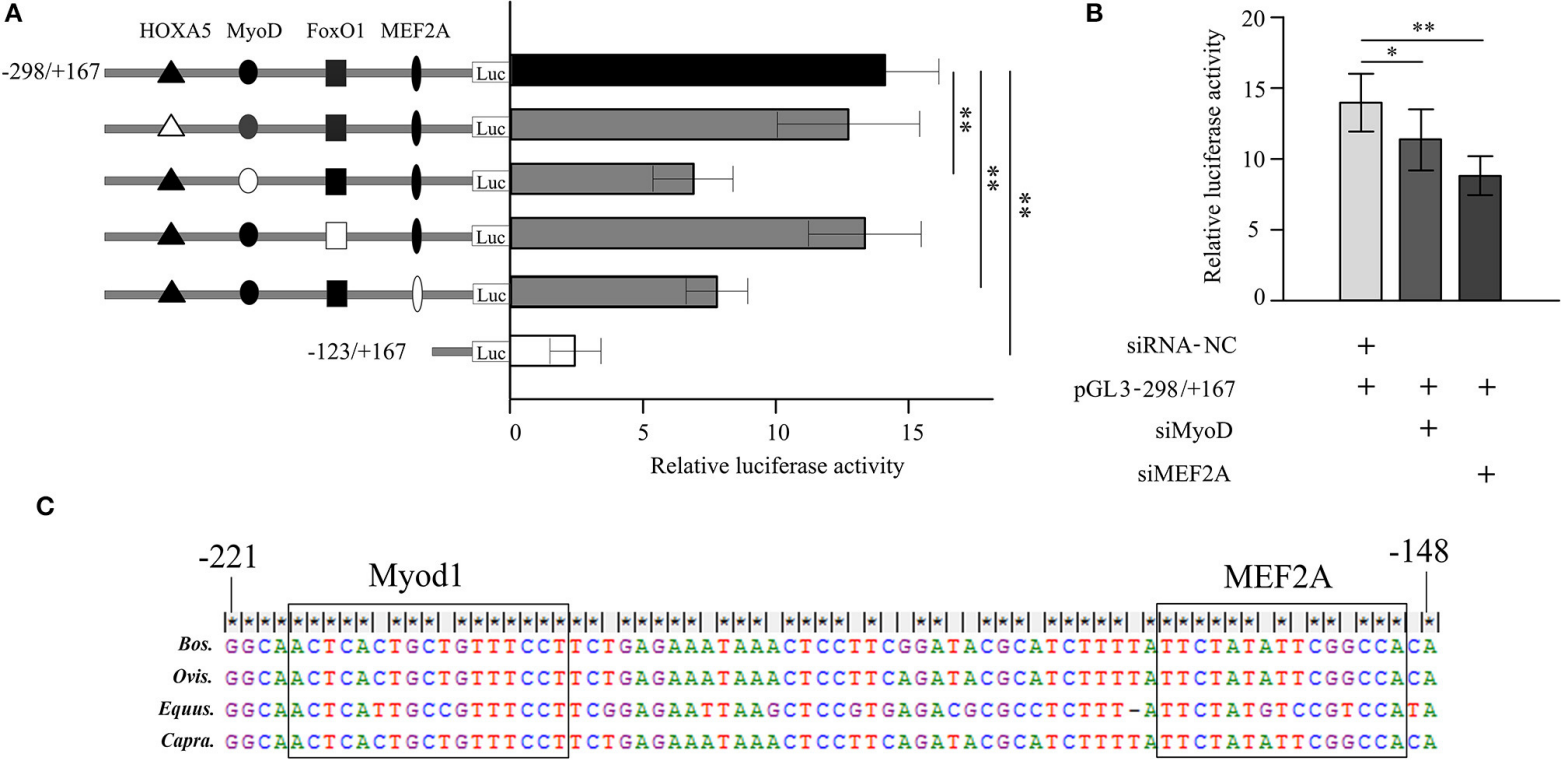
Analysis of transcriptional activity for the corresponding transcription factor in Qinchuan cattle myoblast cells (QCMCs). **(A)** Site-directed mutagenesis for homeobox A5 (HOXA5), myogenic differentiation 1 (Myod1), forkhead box O1 (FoxO1), and myocyte enhancer factor 2A (MEF2A) sites were carried out in the construct pGL−298/+167. The correspondence constructs were transiently transfected into QCMCs and pGL−123/+167 construct was used as a negative control. **(B)** Myod1 and MEF2A knockdown by small interfering RNA (siRNA) co-transfected with pGL−298/+167 in QCMCs. The NC siRNA was used as a negative control. The results are expressed as the means ± SD in arbitrary units based on the firefly/*Renilla* luciferase activity for triplicate transfections. **(C)** Multialignment sequence analysis of the Myod1 and MEF2A transcription factor binding sites in the promoter region of the *LATS*1 gene in cattle, sheep, horse, and goats. “*” *p* < 0.05 and “**” *p* < 0.01.

Many transcription factors contained homeobox A5 (HOXA5), myogenic differentiation 1 (Myod1), forkhead box O1 (FoxO1), and myocyte enhancer factor 2A (MEF2A) were predicted binding in the core promoter region (−298/−123 bp) of bovine *LATS*1 gene by using Genomatix software. To determine the effects of these transcriptional sites in the regulation of *LATS*1, 4-bp point mutations in the HOXA5, Myod1, FoxO1, and MEF2A motifs in p*LATS*1–298/+167 plasmids were created and transfected into QCMCs. As shown in Figure 5A, mutation of the Myod1 and MEF2A site can remarkably decrease the activities of the construct p*LATS*1–298/+167 to 48.9 (*p* < 0.01) and 43.9% (*p* < 0.01) compared to the control group, respectively. However, there was no significant difference (*p* > 0.05) in inhibiting or simulating after the HOXA5- and FoxO1-binding site mutation.

To further verify the role of these potential TFs in the transcriptional process, siRNA-Myod1 and siRNA-MEF2A were co-transfected with p*LATS*1–298/+167 plasmids. The results showed that transiently expressed siRNA could specifically interfere with the transcription levels of *LATS*1 that decreases by 20.4 (*p* < 0.05) and 38.9% (*p* < 0.01) (Figure 5B). In addition, we determined the conservation of Myod1 and MEF2A elements in different species, such as in cattle, sheep, horse, and goat (Figure 5C). These results indicated that Myod1 and MEF2A motifs play an important role as binding sites for Myod1 and MEF2A TFs in regulation of *LATS*1 transcriptional activity.

### Myogenic Differentiation 1 and Myocyte Enhancer Factor 2A Bind to the Promoter Region

**Figure 6:**
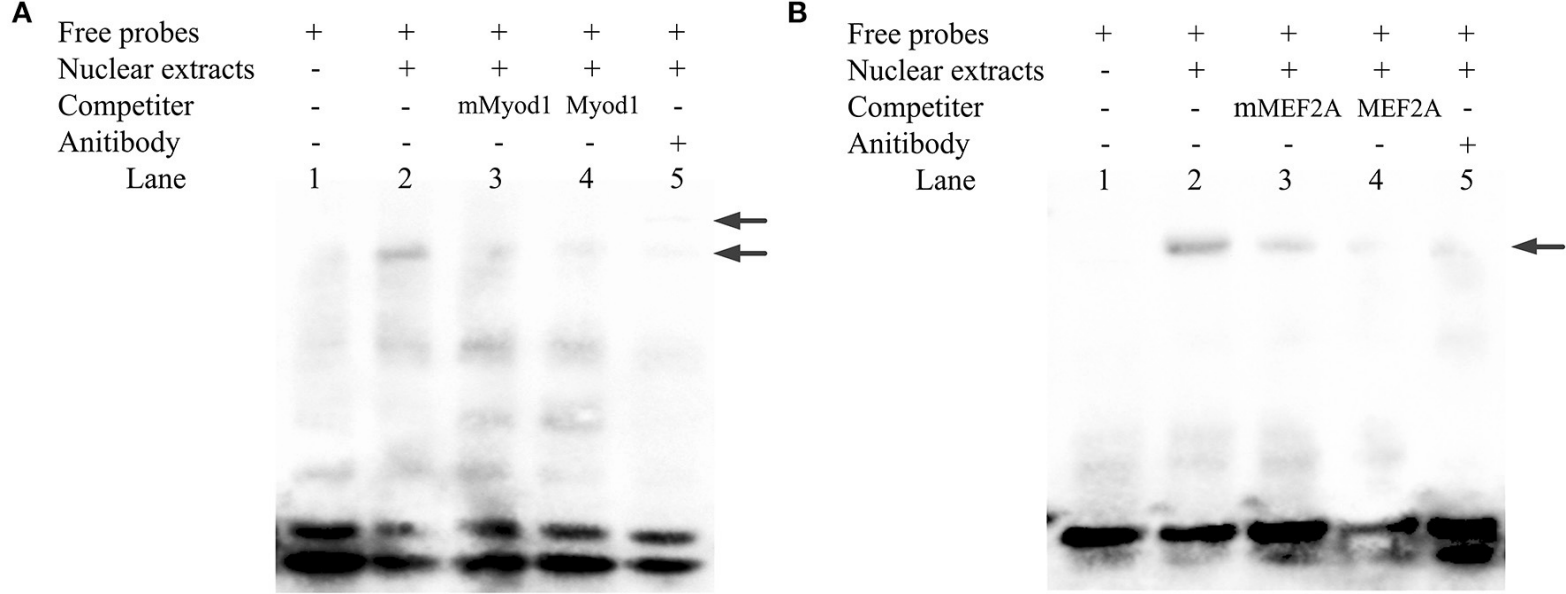
Identification of Myod1 and MEF2A bind to the proximal minimal promoter of *LATS*1 by electrophoretic mobility shift assay (EMSA) *in vitro*. **(A,B)** Nuclear protein extracts were incubated with 5′-biotin-labeled probe containing the Myod1- and MEF2A-binding site in the presence or absence of competitor (lane 2), 50X mutation probe (lane 3), and 50X unlabeled probes (lane 4). The supershift assay was conducted using 10 μg of anti-Myod1 and anti-MEF2A antibodies (lane 5). The arrows mark the main complexes and supershifted, respectively.

Electrophoretic mobility shift assays experiment was performed to determine the binding of Myod1 and MEF2A on the *LATS*1 core promoter. As shown in Figures 6A,B, the QCMCs nuclear protein bound to the 5'-biotin-labeled Myod1/MEF2A probes and formed one main complex (lane 2, Figures 6A,B). When unlabeled wild and mutant types of Myod1/MEF2A were mixed, an upshifted band resembling the complex was barely visible (lane 3–4, Figures 6A,B). Furthermore, the addition of anti-Myod1/MEF2A antibody led to a strong decrease in the upshifted band (lane 5, Figures 6A,B). However, the EMSA experiment of MEF2A revealed no supershifted product at the binding sites. The results suggested that the supershift may be formed by a high-molecular weight polymer and be stuck in the top of the well, causing reduction in gel mobility shift (lane 5, Figure 6B).

**Figure 7:**
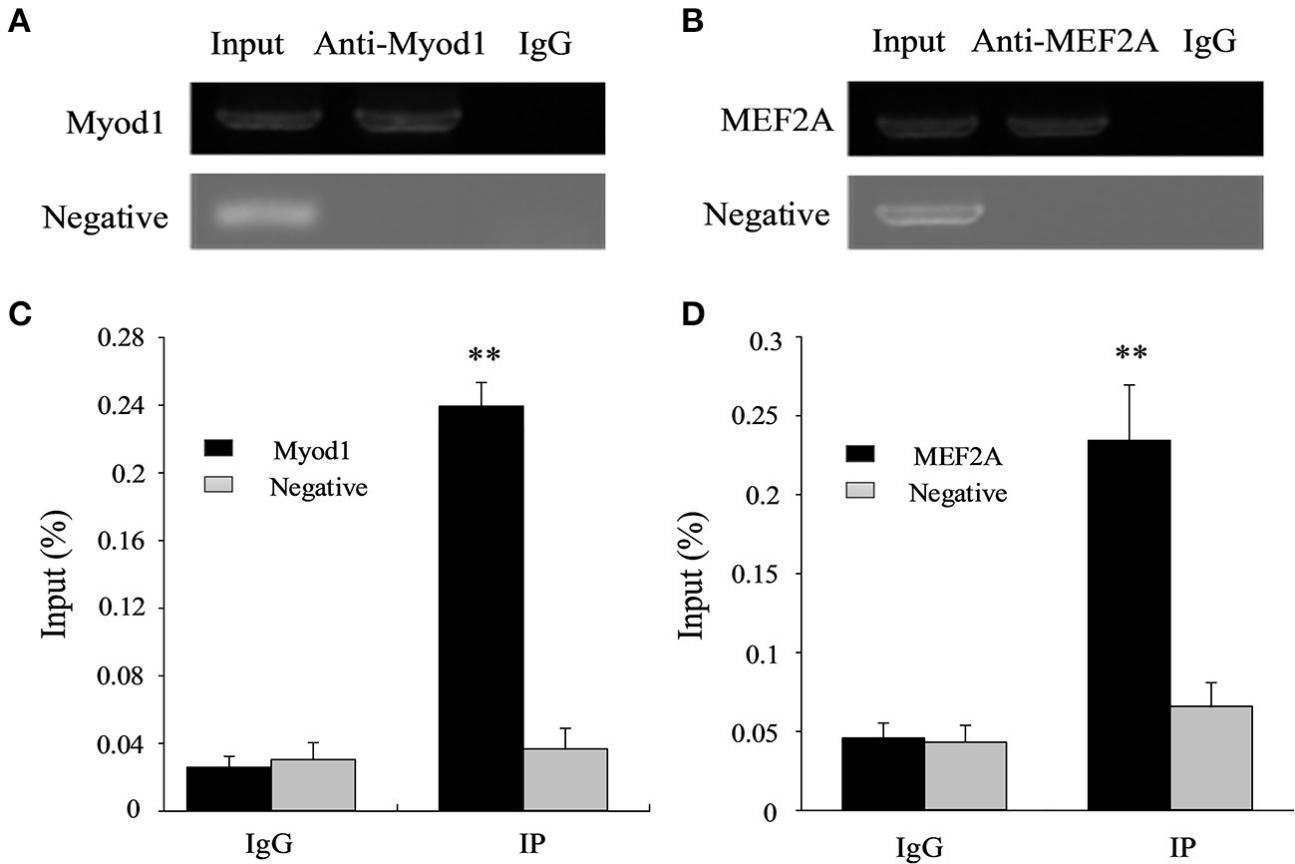
Chromatin immunoprecipitation (ChIP) assay of Myod1 and MEF2A binding to the *LATS*1 promoter *in vivo*. ChIP-PCR products of Myod1 **(A)** and MEF2A **(B)** input and immunoprecipitated were amplified and identified by 1% agarose electrophoresis from muscle. ChIP-qPCR assays detected the enrichment of DNA fragments in samples immunoprecipitated with Myod1 **(C)** and MEF2A **(D)** antibodies. We used the input was total chromatin from muscle, while the products from rabbit antiimmunoglobulin G (IgG) and an intragenic DNA fragment of *LATS*1 exon 3. “**” *p* < 0.01.

Further, a ChIP experiment was carried out to test whether Myod1 and MEF2A can be combined in the corresponding region of the *LATS*1 *in vivo*. The results showed that the relative enrichment levels was ~9.2- and ~5.2-fold over the immunoglobulin G (IgG) control, respectively (Figure 7). These results suggest that Myod1 and MEF2A TFs bind to the promoter region *in vitro* and *vivo* and play an important role in regulating the transcriptional activity of the *LATS*1 gene.

## Discussion

The skeletal muscle is a complicate and dynamic tissue and directly affects the efficiency and quality of animal production. Transcription factors are the key components in the transcription machinery of specific genes, while many transcription co-factors combine with the transcription factors to orchestrate the whole transcription event. The induced expressions of many genes are controlled by specific transcription factors and *cis-*acting elements (19, 22, 23). As a downstream target of the Hippo pathway, *LATS*1 plays a significant part in myoblast proliferation, atrophy/hypertrophy regulation, and transferring mechanical signals into transcriptional responses (24, 25). Recent studies have found that the *LATS* gene family cannot only regulate the proliferation and differentiation of cancer cells, but also regulate muscle growth and development of animals (26–29). In this study, it was found that *LATS*1 gene was highly expressed in the liver, brain, *longissimus dorsi*, large intestine, and abdominal fat tissues of cattle and its protein was highly conserved in the evolution of different species. *LATS*1 gene plays an important role in the process of individual growth and development.

In this study, the promoter sequence of the bovine *LATS*1 gene was obtained and two CpG islands in its promoter 2-kb were found by online software analysis. Approximately half of the genes in vertebrates have CpG-rich sequences on their promoters, namely, CpG islands (CGIs). Studies have shown that a large class of CGIs are located near the transcriptional regulatory region, especially remote from annotated transcription start sites (TSSs). DNA methylation usually occurs in the promoter and exon 1 CGIs genome sequence and it can regulate gene transcription (30–32). It is generally believed that methylation of CGIs can inhibit gene transcription by binding with transcription factors or changing chromatin structure (30, 31). Therefore, we speculated that *LATS*1 was essential in epigenetic modification during the development of individual embryos or tissues and organs.

The core region of *LATS*1 gene was detected by the method of deletion of double luciferase activity step by step and it was found that the core region was located in −298/−123 bp. After analysis with online prediction software, we found the potential transcription factor-binding sites Myod1 and MEF2A on the sequence from nucleotide −221 to −148 bp to be conserved in domestic animals. Myod1 is a member of the myogenic regulatory factor (MRFs) family (33, 34) and plays an important regulator of muscle cell differentiation and muscle fiber formation. It can be involved in the proliferation, differentiation, and regeneration of muscle cells (35). The expression of Myod1 gene regulated the growth and differentiation of muscle and affected the quality of meat (36, 37). In addition, it was found that the overexpression of Myod1 gene was positively correlated with the percentage of type II B fiber in the muscle development, indicating that Myod1 may have the potential function of regulating muscle differentiation in the process of secondary myocyte formation in muscle development (38, 39). Many studies have shown that Myod1 gene plays an important role in muscle growth and development. In this study, we determined Myod1 binding to the core promoter of bovine *LATS*1 gene by using EMSA and ChIP, which suggested that Myod1 plays an important role in regulating the transcription of *LATS*1 gene.

**Figure 8:**
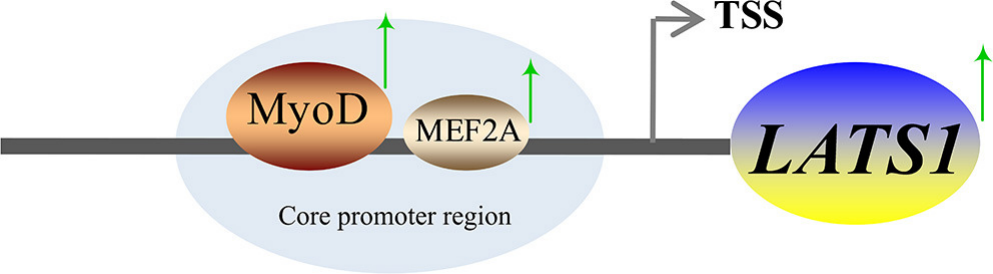
A proposed schematic summary of the regulation of *LATS*1 expression by Myod1 and MEF2A transcription factors (TFs).

Myocyte enhancer factor 2A is a member of the MEF2 gene family, which also includes MEF2B, MEF2C, and MEF2D (40). MEF2A is a transcription factor with the basic helix-loop-helix domain and it is abundant in the brain, skeletal muscle, and myocardium. It plays an important role in promoting muscle fiber formation, muscle fiber homeostasis, cardiomyocyte proliferation, differentiation, and regeneration of skeletal muscle stem cells and myoblasts (17, 41, 42). The latest study found that bovine MEF2A gene silencing inhibited myoblast differentiation and significantly downregulated Myozenin 2 expression (17). To sum up, MEF2A plays an important role in regulating muscle growth and development. In this study, the mutation and knockdown of MEF 2A decreased the activity of basal promoter of *LATS*1. The EMSA and ChIP results showed that MEF2A was capable of high affinity to *LATS*1, thereby suggesting that MEF2A plays an important role in regulating the transcriptional activity of the *LATS*1 gene. A summary is shown in Figure 8 and clarifies the mechanism of transcriptional regulation of bovine *LATS*1 gene.

## Conclusion

In summary, this study identified that the relative expression of bovine *LATS*1 gene was highly expressed in lung, kidney, heart, subcutaneous fat, and *longissimus dorsi* muscle. The core region of bovine *LATS*1 gene was located at −298/−123 bp and is regulated by Myod1 and MEF2A TFs. These results laid a theoretical foundation for exploring the molecular regulation mechanism of the bovine *LATS*1 gene in muscle growth.

## Data Availability Statement

The original contributions presented in the study are included in the article further inquiries can be directed to the corresponding author/s.

## Ethics Statement

The animal study was reviewed and approved by all animal operating procedures are in line with the China Council on Animal Care, as well as the standards set by the Ningxia University of experimental animal management practices.

## Author Contributions

DW and SR designed the experiment performed the experiments and wrote the manuscript. XW, RK, MAla, and BA mainly assisted in analyzing the data. AAls, AAb, ZL, JZ, and YM provided constructive suggestions for the discussion. GZ, DW, and ZL contributed in project administration and supervision. MAlh and AAla contributed in analysis data, validation, writing—review, and editing. All authors have read and agreed to the published version of the manuscript.

## Funding

This study was supported by the Natural Science Foundation of China (32060744 and 31960672), the Natural Science Foundation of Ningxia (2021AAC05007 and 2020AAC03079), the Key R&D Projects in Ningxia Hui Autonomous Region (2019BEF02004 and 2020BEF02011), the Top Discipline Construction Project of Pratacultural Science of Ningxia University (NXYLXK2017A01), and the Ph.D., Research Startup Foundation of Ningxia University (030900001926).

## Conflict of Interest

The authors declare that the research was conducted in the absence of any commercial or financial relationships that could be construed as a potential conflict of interest. The reviewer CM declared a shared affiliation with the author SR to the handling editor at the time of the review.

## Publisher's Note

All claims expressed in this article are solely those of the authors and do not necessarily represent those of their affiliated organizations, or those of the publisher, the editors and the reviewers. Any product that may be evaluated in this article, or claim that may be made by its manufacturer, is not guaranteed or endorsed by the publisher.
